# Exploring dietary changes and supplement use among cancer patients in Norway: prevalence, motivations, disclosure, information, and perceived risks and benefits: a cross sectional study

**DOI:** 10.1186/s40795-024-00872-8

**Published:** 2024-04-26

**Authors:** Agnete E. Kristoffersen, Trine Stub, Jorunn V. Nilsen, Johanna Hök Nordberg, Ann Ragnhild Broderstad, Barbara Wider, Mona Bjelland

**Affiliations:** 1 National Research Center in Complementary and Alternative Medicine (NAFKAM), Department of Community Medicine, UiT The Arctic University of Norway, Tromsø, Norway; 2 The Norwegian Cancer Society, Oslo, Norway; 3 Regional Cancer Center Stockholm Gotland, Stockholm, Sweden; 4 Department Neurobiology, Care Sciences & Society, Division of Nursing & Department Physiology & Pharmacology, Karolinska Institutet, Stockholm, Sweden; 5 Center for Sami Health Research, UiT The Arctic University of Norway, Tromsø, Norway; 6 Clinic of Medicine, University Hospital of North Norway, Harstad, Norway

**Keywords:** Nutrition, Cancer, Diet, Dietary supplements

## Abstract

**Background:**

Cancer is the leading cause of death in Norway, with prostate, breast, lung, and colon cancers being the most prevalent types. Adopting a healthy and varied diet can help reduce cancer risk and recurrence. However, access to dietary counselling remains limited for cancer patients in Norway. This study aimed to investigate the prevalence of dietary supplement use and dietary changes made by cancer patients and survivors. Additionally, it sought to explore the reason(s) for such practices, communication with healthcare providers, sources of information, and reported benefits and potential harms resulting from these changes and supplement use.

**Methods:**

Conducted in collaboration with the Norwegian Cancer Society (NCS), this online cross-sectional study targeted members of their user panel who had either current or previous cancer (*n* = 706). The study took place in September/October 2021, utilizing a modified cancer-specific version of the International Questionnaire to Measure Use of Complementary and Alternative Medicine (I-CAM-Q). Out of 468 participants (315 women and 153 men), 67.2% consented to participate. Between-group analyses were conducted using Pearson chi-square tests and Fisher exact tests for categorical variables, while independent sample t-tests were applied for continuous variables.

**Results:**

The majority of the participants (97%) reported making changes to their diet (78%) and/or incorporating dietary supplements (73%) in response to their cancer diagnosis. The primary goal of these changes was to strengthen their body and immune system. Almost half of the participants (49%) reported that they found these changes beneficial and discussed them openly with their healthcare providers, with family physicians being the most common point of discussion (25%). Adverse effects were reported by only a few participants, mostly mild. Information about dietary changes and supplements was primarily sourced from the internet or healthcare providers.

**Conclusions:**

This study highlights that most individuals affected by cancer attribute to dietary adjustment. It also emphasizes the importance of addressing adherence to dietary recommendations and using reliable sources of information. Additionally, the study highlights the potential, yet currently underutilized, role of healthcare professionals in initiating dialogues about dietary interventions to address any unmet needs of patients. Such proactive engagement may contribute to the promotion of reliable sources of information and the prevention of non-evidence-based and potentially harmful diets or supplement adoption.

## Background

Cancer stands as the leading cause of death in Norway, accounting for 25% of all deaths in 2022, resulting in 11,282 fatalities [1]. Approximately 38,000 receive a cancer diagnosis annually with a mean survival rate of 75%. A higher percentage of men (54%, *n* = 20,487) are diagnosed with cancer compared to women (46%, *n* = 17,778). The most prevalent cancer types in Norway include prostate cancer (14%, *n* = 5474), breast cancer (11%, *n* = 4247), lung cancer (9%, *n* = 3534), and colon cancer (8%, *n* = 3252) [2]. As of the conclusion of 2022, there were 327,101 cancer survivors living in Norway [2].

A healthy and varied diet is known to reduce the risk of cancer and its recurrence [3]. To achieve this, experts recommend consuming plenty of whole grains, vegetables, legumes, fruits, and fish, along with incorporating low-fat dairy products [4–9]. It is also advisable to limit the intake of processed foods, red meat, and food and drinks high in salt, sugar, and fat. Staying hydrated with water when thirsty, limiting alcohol consumption, and maintaining a healthy body weight are essential [7–10]. During and after cancer treatment for head and neck, lung, and gastrointestinal cancers, patients have reported experiencing unintentional weight loss due to the adverse effects of treatment or the impact of cancer itself [11]. Conversely, weight gain is commonly observed during and after breast cancer treatment, often attributed to chemotherapy and endocrine therapies, induced menopause, changes in metabolism and food intake, and decreased physical activity [12].

Diet plays a crucial role in the lives of cancer patients and their families, offering a controllable aspect that can be adjusted during the course of cancer treatments [13]. Research has highlighted that cancer patients express a strong desire for dietary advice and guidance [14, 15]. Nevertheless, in Norway, access to dietary counseling remains limited, especially in regions outside the south-eastern area, centered around the capital, Oslo [16]. The potential impact of various dietary interventions and supplements on cancer therapy is currently under investigation [17], as many patients are intrigued by the possibility that these changes may enhance treatment response, minimize drug-induced adverse effects, or improve overall quality of life [18]. While these hypotheses are appealing, more research is needed to fully understand the relationships between diet, dietary supplements, cancer therapy, and cancer itself. Meanwhile, it is crucial to recognize that certain restrictive diet regimens and dietary interventions may pose both direct and indirect risks for patients [19, 20]. However, guidelines have been developed to assist healthcare professionals in advising patients on these matters [21].

Dietary changes among cancer patients and survivors, in accordance with recommendations, seem to be widespread and consistent across different countries and over time [22–25]. These changes often include increasing the intake of fibre, fruits, and vegetables while reducing the consumption of meat, fat, and sugary foods like desserts [22–25]. However, despite these guidelines, adherence to the ‘five-a-day’ recommendation (consuming five different fruits and vegetables daily) is reported to be low among Norwegian cancer patients [26].

Cancer patients commonly cite several main reasons for making dietary changes after their diagnosis and treatment. These include the desire to prevent cancer recurrence, support therapy and recovery, improve overall health, and manage adverse effects related to the cancer diagnosis and treatment [14].

The use of dietary supplements is frequently reported by cancer patients, ranging from 7 to 66% across European countries [27]. Dietary supplements are products intended to supplement the diet with concentrated sources of vitamins, minerals, or other substances with nutritional or physiological effects, either alone or in combination. They are available in prepackaged, measured forms, such as capsules, lozenges, tablets, pills, powder bags, ampoules, dropper bottles, and similar liquid and powder forms [28]. Around 70% of the general Norwegian population utilizes dietary supplements, and this use has remained consistent over the past decade [29]. These supplements are more commonly used by younger and highly educated individuals, with women using them more frequently than men [29]. The most reported reason for supplement use is to enhance well-being [22–24, 30–33]. However, it is essential to exercise caution when incorporating dietary changes and using supplements without guidance from a nutritionist, as they may potentially interact negatively with cancer treatment. For this reason, researchers recommend open communication between patients and healthcare providers regarding dietary changes and supplement use [22–24, 30–32].

The aim of this study was to examine to what degree cancer patients and survivors use dietary supplements and how they adjust their diets after being diagnosed with cancer. Additionally, the study sought to understand the reasons behind these choices, the extent of communication with healthcare providers, where individuals gather information, and the perceived advantages and potential risks associated with dietary changes and supplement use.

## Methods

In close cooperation with the Norwegian Cancer Society (NCS), we conducted an online cross-sectional study among the members of their user panel who currently have or previously have had cancer (*n* = 706). The study was carried out in September/October 2021 using a cancer-specific version of the International Questionnaire to Measure Use of Complementary and Alternative Medicine (I-CAM-Q) [34].

### Participants

Members of the NCS’s user panel, aged 18 years or above with a current or past cancer diagnosis were invited to participate in the survey. The NCS’s user panel consists of a higher proportion of women (75%) than men (25%). The majority of panel members fall within the age range of 50 to 69 years old. Recruitment of members primarily takes place through social media, the NCS’s webpage, and social events.

### Recruitment and data collection

**Fig. 1:**
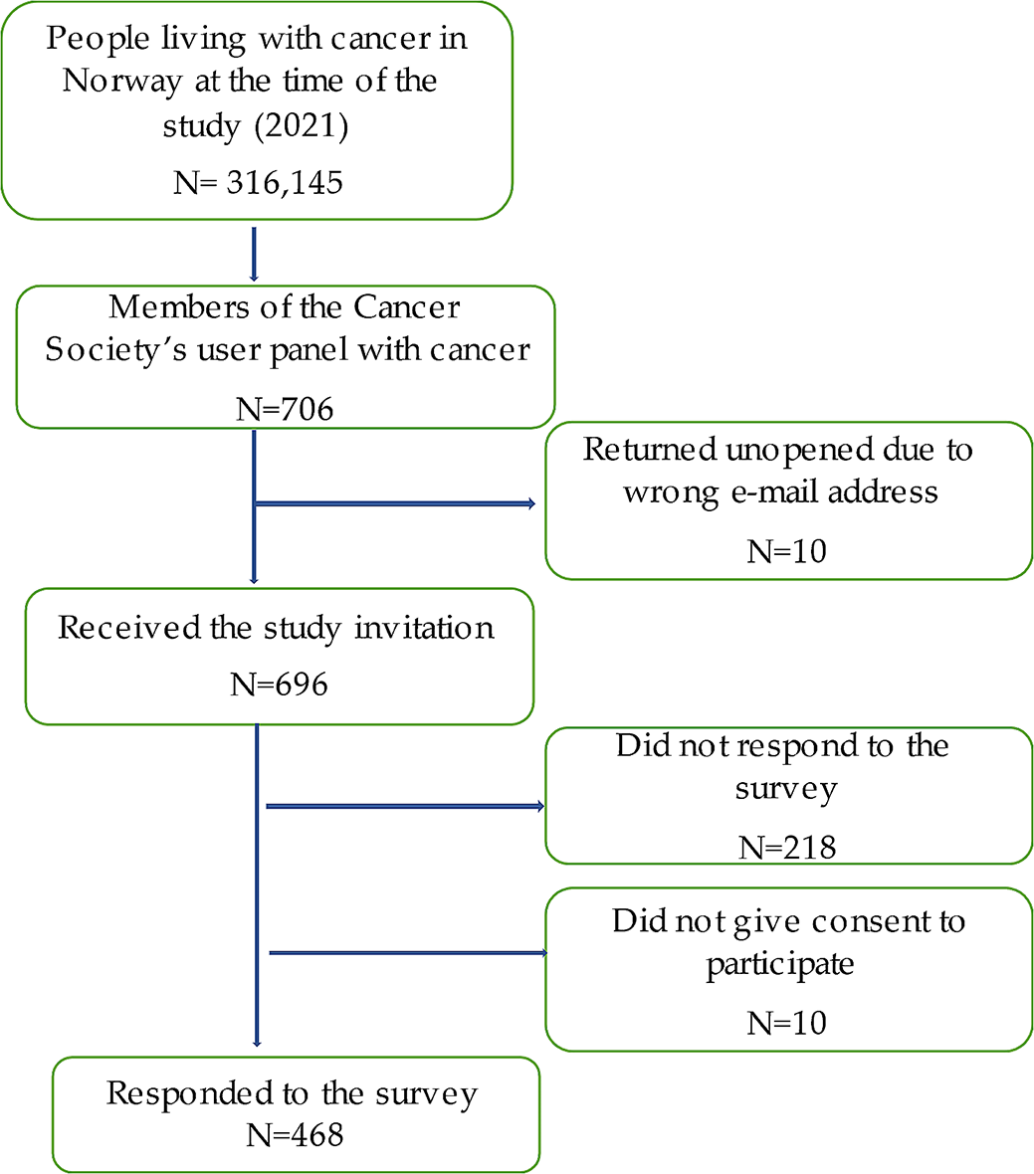
Flow chart of the included participants

All members of the user panel who had current or past cancer (*n* = 706) received an invitation via email from the NCS, which included a link to the survey. To proceed to the survey, participants had to give their consent to participate by ticking a box in the information letter. The survey was exclusively conducted online. Out of the 696 members who received the invitation, 478 responded, and 468 provided their consent to participate, resulting in a response rate of 67.2% (Fig. 1).

### Measures

The survey was based on a cancer specific version of the 

 I-CAM-Q questionnaire [34] and included questions about dietary changes and supplement use, visits to complementary and alternative medicine (CAM) providers, self-help practices, physical activity, and spiritual practices, of which dietary changes and supplement use are reported in this current study (Tables 2 and 3 for complete list of dietary changes and supplements asked). Visits to CAM providers, use of self-help practices, physical activity and spiritual practices are reported elsewhere [18].

Upon enrollment, the NCS had already acquired age, gender, and cancer diagnosis data for all user panel members, which were subsequently integrated into the survey questions for all participants. Additionally, data on income and education were collected during the study.

For all dietary changes and supplements utilized, the panel members were asked follow-up questions regarding the reason(s) for making these alterations or using the supplements: (1) *To treat/slow down the cancer or prevent the cancer from spreading*; (2) *Treat adverse effects / late and long-term effects of the cancer or cancer treatment*; (3) *Strengthen the body / immune system*; (4) *Increase quality of life, coping, relaxation or well-being*; (5) *Other reasons*), and possible adverse effects of these approaches (without specification of the adverse effect): (1. *Yes, serious*; 2. *Yes, moderate*; 3. *Yes, mild*; 4. *No*; 5. *Do not know*). Regarding the type of dietary changes or supplements used, participants were asked to express their experiences related to the potential effects of these interventions. Participants were presented with the following options: (1) *Experienced that I got better*; (2) *No change*; (3) *Got worse*; and (4) *Do not know*. Additionally, participants were inquired about the sources from which they obtained information about these approaches, with the following response categories: (1) *Internet / media*; (2) *Health personnel (doctor / nurse etc.)*; (3) *CAM provider*; (4) *Friends, family etc.*.; (5) *Other*; (6) *Do not remember / do not know*; (7) *Did not receive / did not seek information*. Furthermore, participants were asked whether they had discussed these dietary changes or supplement use with the following: (1) *Family physician*; (2) *Oncologist*; (3) *Nurse*; (4) *Other health personnel (nutritionist etc.)*; (5) *CAM provider*; (6) *None of these*; (7) *Do not remember / do not know*.

### Measures of personal characteristics

Age was collected as an open question and later categorized into three groups: *19–50 years*, *51–64 years*, and *65 - 82 years*, to be used both as a continuous variable and a categorical variable.

Level of education was gathered using four categories: (1) *Primary school up to 10 years’ duration*; (2) *Secondary school of 10–12 years’ duration*; (3) *College/university less than 4 years’ duration*; and (4) *College/university 4 years’ duration or more*.

Household income was collected using the following categories *NOK < 400,000 (low income)*; *NOK 400,000-799,000 (medium income)*, and *NOK 800,000 or more (high income).* Additionally, participants had the option not to provide income information.

Other personal characteristics considered were gender (*male, female*) and place of residence, which were consolidated into the following Norwegian regions: *South-East*, *South*, *West*, *Central (Trøndelag)*, and *North*.

### Statistics/ power calculation

To achieve an adequate study power, with a margin of error of 5%, a confidence level of 95%, and a heterogeneity of 50%, a minimum sample of *n* = 384 was required to accurately represent the Norwegian cancer population of 316,145 at the time of the study (2021) [35]. Descriptive statistics were performed using frequency analyses and cross-tabulation. Between-group analyses involved the use of Fisher exact tests and Pearson chi-square tests for categorical variables, while independent sample t-tests were applied for continuous variables. The significance levels were set at *p* < 0.05. All analyses were conducted using SPSS V.29.0 for Windows.

## Results

### Participant characteristics: general and disease-specific information

The NCS’s user panel is predominantly composed of women (75%) compared to men (25%), leading to a higher proportion of women (67%) than men (33%) in the study (*p* < 0.001). The mean age of female participants was 57.3 years, while male participants had a mean age of 62.9 years (*p* < 0.001).

Most of participants had attained college or university education (63%) and reported high income levels (46%). Geographically, the largest proportion of participants (52%) resided in the South-Eastern part of Norway in the vicinity of the capital, Oslo.

Living arrangements also varied between genders, with more men (75%) than women (63%, *p* = 0.008, Table 1) residing with a spouse/partner.

**Table 1:** Participant characteristics and associations with cancer-related dietary changes and dietary supplement use

	Total		Women		Men			Dietary changes			Dietary supplements		
	%	*n* = 468	%	*n* = 315	%	*n* = 153	p-value	%	*n* = 373	p-value	%	*n* = 340	p-value
**Sex**										0.240*			0.307*
Women	67.3	315						80.1	246		78.0	234	
Men	32.7	153						84.7	127		73.6	106	
**Age**							< 0.001*			0.675*			0.035*
19–50 years	23.1	100	27.9	81	13.3	19		83.0	83		67.0	67	
51–64 years	41.3	179	43.4	126	37.1	53		79.3	142		80.4	144	
65–82 years	35.6	154	28.6	83	49.7	71		82.5	127		77.8	119	
Mean age years (SD)	59.18 (11.295)		57.34 (11.277)		62.92 (10.408)		< 0.001**	58.98 (11.455)		0.444**	59.43 (11.420)		0.889**
**Education**							0.319*			0.223*			0.502*
Primary school	6.5	28	5.2	15	9.1	13		71.4	20		67.9	19	
Secondary school	30.3	131	29.3	85	32.2	46		85.5	112		77.7	101	
College/ University < 4 years	33.9	147	35.9	104	30.1	43		78.2	115		74.1	109	
College/ University ≥ 4 years	29.3	127	29.7	86	28.7	41		82.7	105		79.5	101	
**Household income**							0.477*			0.937*			0.482*
Low (Less than EUR 40 000)	10.4	45	10.3	30	10.5	15		80.0	36		75.6	34	
Middle (EUR 40 000 to 79 000)	35.1	152	35.9	104	33.6	48		80.3	122		72.4	110	
High (EUR 80 000 or more)	46.4	201	44.5	129	50.3	72		82.6	166		79.5	159	
No reply	8.1	35	9.3	27	5.6	8		80.0	28		77.1	27	
**Household** ^**1**^													
Live alone	20.7	97	22.9	72	16.3	25	0.103*	80.4	78	0.730*	77.3	75	0.845*
Live with a partner	66.9	313	62.9	198	75.2	115	0.008*	81.5	255	0.903*	76.6	239	0.984*
Live with own children	18.2	85	21.3	67	11.8	18	0.012*	78.8	37	0.461*	68.2	58	0.043*
Other	1.5	7	1.6	5	1.3	2	1.000^	100	7	0.359^	71.4	5	0.668^
**Place of residence (region)**							0.460*			0.041^			0.384^
South-East	51.7	242	53.3	168	48.4	74		79.9	191		73.7	171	
South	4.3	20	4.1	13	4.6	7		70.0	14		80.0	16	
West	24.8	116	22.5	71	29.4	45		83.9	94		77.1	84	
Central (Trøndelag)	8.5	40	8.3	26	9.2	14		75.0	27		77.8	28	
North	10.7	50	11.7	34	8.5	13		94.0	47		87.2	41	
**Cancer site** ^**1**^													
Breast	39.1	183	57.8	182	0.7	1	< 0.001*	75.4	135	0.006*	77.5	134	0.726*
Gastro intestinal	13.7	64	10.5	33	20.3	31	0.004*	87.3	55	0.210*	80.3	49	0.456*
Male genitalia	11.1	52	-	-	34.0	52	-	84.3	43	0.598*	66.7	32	0.086*
Female genitalia	8.1	38	12.1	38	-	-	-	84.2	32	0.667*	94.6	35	0.007*
Lymphoma	8.8	41	6.3	20	13.7	21	0.008*	78.0	32	0.536*	82.9	34	0314*
Malignant melanoma	4.7	22	4.4	14	5.2	8	0.707*	90.9	20	0.249*	59.1	13	0.047*
Head and neck	3.8	18	1.6	5	8.5	13	< 0.001*	83.3	15	0.848*	82.4	14	0.773^
Lung	3.2	15	2.5	8	4.6	7	0.268^	78.6	11	0.765*	85.7	12	0.536^
Sarcoma	3.0	14	3.8	12	1.3	2	0.160^	78.6	11	0.765*	78.6	11	1.000^
Leukemia	2.4	11	2.2	7	2.6	4	0.755^	90.9	10	0.421*	72.7	8	0.724^
Bone marrow	2.1	10	1.9	6	2.6	4	0.735^	100	10	0.129^	70.0	7	0.705^
Other cancer sites	14.3	67	10.8	34	21.6	33	0.002*	90.5	57	0.051*	74.2	46	0.633*
**In active cancer treatment**							0.332*			0.034*			0.277*
Yes	33.8	158	35.2	111	30.7	47		87.0	134		79.6	121	
No	66.2	310	64.8	204	69.3	106		78.9	239		75.0	129	
**Late and long-term effects**							0.045*			0.064*			0.041*
**No**	11.5	50	8.9	26	16.8	24		70.0	35		64.0	32	
**Yes, the following**:	82.9	360	84.9	247	79.0	113		83.1	299		78.6	282	
Fatigue	54.9	257	58.4	184	47.7	73	0.029*	84.0	216	0.129*	80.5	207	0.021*
Sleep disorder	38.5	180	45.7	144	23.5	36	< 0.001*	85.6	154	0.080*	82.2	148	0.020*
Hot flushes	36.3	170	46.0	145	16.3	25	< 0.001*	81.2	138	0.851*	81.8	139	0.042*
Nerve damage (polyneuropathy)	35.3	165	42.2	133	20.9	32	< 0.001*	83.0	137	0.558*	82.4	136	0.025*
Pain	34.0	159	40.0	126	21.6	33	< 0.001*	83.0	132	0.573*	78.6	125	0.448*
Decreased muscle strength and mobility	33.8	158	39.4	124	22.2	34	< 0.001*	83.5	132	0.440*	80.3	126	0.176*
Cognitive challenges	31.8	149	41.0	129	13.1	20	< 0.001*	84.6	126	0.258*	83.2	124	0.019*
Sexual problems	29.3	137	26.7	84	34.6	53	0.075*	83.9	115	0.402*	78.8	108	0.453*
Gained weight	25.2	118	30.8	97	13.7	21	< 0.001*	86.4	102	0.116*	83.9	88	0.028*
Anxiety or depression	20.5	96	22.5	71	16.3	25	0.119*	91.7	88	0.004*	86.5	83	0.010*
Early menopause	19.2	90	28.6	90	-	-	**-**	77.8	70	0.294*	82.2	74	0.157*

* Pearson chi-square test; ^Fisher exact test

** Independent sample t-test

^1^Multiple choice

Among female participants, the most prevalent cancer type was breast cancer (58%), followed by female genitalia cancer (12%) and gastrointestinal cancer (11%). For male participants, the most common diagnoses were male genitalia cancer (34%), gastrointestinal cancer (20%), and lymphoma (14%). Approximately one-third of the participants (34%) were undergoing active cancer treatment at the time of the survey (Table 1).

### Modifications to existing diet and adoption of special diets

Modifying an existing diet usually involves making small adjustments to the foods and nutrients consumed, while keeping the overall framework of the diet intact. This may involve adding or removing certain foods. Compliance with special diets, on the other hand, usually requires a more significant overhaul of the foods and nutrients consumed. This might involve adopting a new eating pattern, such as a vegetarian or Mediterranean diet, or eliminating certain types of foods altogether.

Many of the participants (81.6%) had either made modifications to their diet (79.5%) or switched to special diets (42.3%) because of their cancer diagnosis (Table 2). Among those who made these changes, the primary motivations were to strengthen the body/immune system (72.1%) or to improve quality of life, coping, relaxation, or well-being (63.0%). Approximately half of the participants (48.8%) who changed their diet found one or more of the adjustments to be beneficial. Only a small percentage (13.7%) reported experiencing adverse effects from the dietary changes they made (Table 2). Participants who were in active cancer treatment (*p* = 0.045), dealing with anxiety or depression (*p* = 0.004), and residing in the northernmost part of Norway (*p* = 0.041) were more likely to alter their diet. Conversely, individuals diagnosed with breast cancer were less likely to make changes to their diet (*p* = 0.006, Table 1).

**Table 2:** Dietary changes made by cancer patients: Reasons, benefits, and adverse effects

								Reason(s) for use**													
	**Total**		**Women**		**Men**		**p-value**	**To treat cancer or prevent it from spreading**		**To treat side effects or late effects of cancer/ cancer treatment**		**To strengthen the body / immune system**		**To increase quality of life, coping, relaxation or well-being**		**Other reason**		**Improvement**		**Adverse effects**	
	%	n	%	n	%	n		%	n	%	n	%	n	%	n	%	n	%	n	%	n
**Modifications to exicting diet**	**79.5**	**364**	**78.6**	**242**	**81.3**	**122**	0.492*	**23.9**	**87**	**31.6**	**115**	**69.5**	**253**	**61.3**	**223**	**45.9**	**167**	**45.5**	**164**	**12.1**	**44**
Increased intake of fruit and vegetables	55.7	255	56.5	174	54.0	81	0.614*	12.2	31	12.5	32	78.0	199	48.6	124	13.3	34	-	-	6.3	16
Reduced intake of suger	45.9	210	46.4	143	44.7	67	0.722*	27.1	57	27.1	57	47.1	99	51.0	107	17.6	37	-	-	10.5	22
Increased intake of fish	43.9	201	40.6	125	50.7	76	0.041*	8.0	16	12.9	26	68.2	137	43.8	88	15.4	31	-	-	4.0	8
Reduced intake of alcohol	42.6	195	45.5	140	36.7	55	0.074*	17.9	35	24.1	47	33.8	66	40.0	78	35.4	69	-	-	4.1	8
Increased intake of whole grains	40.0	183	37.3	115	45.3	68	0.101*	8.2	15	15.8	29	63.9	117	45.4	83	19.7	36	-	-	9.3	17
Reduced intake of carbohydrats	26.0	119	27.9	86	22.0	33	0.175*	18.5	22	26.9	32	36.1	43	54.6	65	29.4	35	-	-	12.6	15
Reduced intake of meat	21.8	100	23.1	71	19.3	29	0.366*	18.0	18	23.0	23	31.0	31	49.0	49	29.0	29	-	-	9.0	9
Reduced intake of dairy products	18.8	86	16.6	51	23.3	35	0.081*	12.8	11	27.9	24	24.4	21	43.0	37	38.4	33	-	-	14.0	12
**Special Diets**	**42.3**	**191**	**42.4**	**129**	**41.9**	**62**	0.913*	**17.8**	**34**	**23.6**	**45**	**66.5**	**127**	**56.0**	**107**	**28.3**	**54**	**49.2**	**94**	**9.9**	**19**
Nutrient dense diet ^1^	23.2	105	22.7	69	24.3	36	0.701*	8.6	9	33.3	23	67.6	71	50.5	53	14.3	15	-	-	9.6	10
Organic food ^2^	16.4	74	17.4	53	14.2	21	0.382*	18.9	14	18.9	10	74.3	55	50.0	37	27.0	20	-	-	4.1	3
Low carb diet ^3^	13.5	61	14.8	45	10.8	16	0.244*	23.0	14	40.0	18	39.3	24	62.3	38	31.1	19	-	-	8.2	5
Fasting ^4^	7.1	32	7.9	24	5.4	8	0.333*	21.9	7	28.1	9	40.6	13	65.6	21	28.1	9	-	-	15.6	5
Vegetarian diet ^5^	2.9	13	3.3	10	2.0	3	0.560^	30.8	4	30.0	3	53.8	7	53.8	7	15.4	2	-	-	0.0	0
Ketogenic diet ^6^	2.7	12	2.6	8	2.7	4	1.000^	41.7	5	50.0	4	50.0	6	50.0	6	8.3	1	-	-	16.7	2
Juice diet ^7^ (carrot, beetroot, apricot etc.)	1.8	8	1.7	5	2.0	3	0.814^	62.5	5	50.0	4	88.0	7	50	4	0.0	0	-	-	50.0	4
Budwig diet ^8^	1.3	6	1.3	4	1.4	2	1.000^	100	6	16.6	1	67.0	4	16.6	1	0.0	0	-	-	0.0	0
Vegan diet ^9^	0.4	2	0.7	2	0.0	0	1.000^	50.0	1	50.0	1	0.0	0	0.0	0	0.0	0	-	-	0.0	0
**Total**	**81.6**	**373**	**80.1**	**246**	**84.7**	**127**	0.240*	**24.7**	**92**	**32.7**	**122**	**72.1**	**269**	**63.0**	**235**	**49.6**	**185**	**48.8**	**182**	**13.7**	**51**

*Pearson chi-square test; ^Fisher exact test

** multiple choice; - data not collected

^1^ Food that is high in nutrients but relatively low in calories; ^2^ Foods and drinks produced by methods complying with the standards of organic farming; ^3^ A diet that restricts carbohydrates and is high in protein, fat, and vegetables; ^4^ Abstention from eating and sometimes drinking over a period of time; ^5^ A diet that do not contain meat, poultry, or fish but might include eggs and dairy products; ^6^ A low carb, high fat diet; ^7^ A diet containing only of juices from vegetables and fruits; ^8^ flaxseed oil and cottage cheese, as well as vegetables, fruits and juices. Processed foods, meats, most dairy products and sugar are prohibited; ^9^ A diet without any products derived from animals

#### Modifications to existing diet

The most common dietary adjustments were made to existing diets (79.5%), with the primary modifications being an increased intake of fruits and vegetables (55.7%), followed by reduced sugar consumption (45.9%) and increased fish intake (43.9%). Regarding gender differences in dietary changes, few were observed. However, men were more likely than women to increase their fish intake (50.7% vs. 40.6%, *p* = 0.041, Table 2). Approximately half of the participants who made changes to their diets reported experiencing improvements after implementing the alterations (45.5%). A small percentage (12.1%) experienced adverse effects, mainly associated with reductions in dairy products (14%), carbohydrates (12.6%), and sugar (10.5%, Table 2). Although most adverse effects were moderate or mild, severe adverse effects were also reported.

#### Special diets

Among the special diets used by participants, the most common was the nutrient-dense diet (23.2%), followed by a diet primarily based on organic food (16.4%) and a low-carb diet (13.5%). The primary reasons for adopting these special diets were to strengthen the body/immune system (66.5%) and to improve quality of life, coping, relaxation, or well-being (56%). There were no significant gender differences in the utilization of these diets (*p* = 0.913), as they were equally embraced by both men and women. About half of the participants (49.2%) who followed special diets reported finding one or more of the diets helpful. Few participants (9.9%) experienced adverse effects, with the most notable being associated with juice diets (50%), ketogenic diets (16.7%), and fasting (15.6%, Table 2).

### Use of dietary supplements

Use of dietary supplements for cancer related complaints were reported by 76.6% of the participants, with 78% of women and 73.6% of men engaging in such practices (*p* = 0.307). The use of dietary supplements was found to be positively associated with being middle-aged (51–64 years, *p* = 0.035) and negatively associated with having children living in the household (*p* = 0.043). Among the various dietary supplements used, vitamins and minerals were the most common (70%), primarily utilized to strengthen the body and the immune system (78.9%, *n* = 247). Additionally, some participants used these supplements to address adverse or late effects of cancer (36.7%, *n* = 115), or enhance their quality of life (26.2%, *n* = 82). A smaller proportion (13.7%, *n* = 43) used supplements specifically to treat cancer or prevent its spread. The most frequently used vitamins were vitamin D (51.2%), multivitamins (27.5%), vitamin B (22.1%), and vitamin C (20.8%). A total of 33.8% of participants reported experiencing improvements after using vitamins and minerals, while 4.5% reported adverse effects (Table 3). Among the adverse effects, one was considered serious, eight were moderate, and five were mild.

**Table 3:** Dietary supplements used by cancer patients: Reasons, benefits, and adverse effects

								Reason(s) for use**													
	Total		Women		Men		p-value	To treat cancer or prevent it from spreading		To treat side effects or late effects of cancer (treatment)		To strengthen the body / immune system		To increase quality of life, coping, relaxation or well-being		Other reason(s)		Improvement		Adverse effects	
	%	n	%	n	%	n		%	n	%	n	%	n	%	n	%	n	%	n	%	n
**Vitamins and minerals**	**70.0**	**313**	**72.7**	**218**	**64.6**	**95**	0.081*	**13.7**	**43**	**36.7**	**115**	**78.9**	**247**	**26.2**	**82**	**16.6**	**52**	**33.,5**	**105**	**4.5**	**14**
Vitamin D	51.2	229	56.0	168	41.5	61	0.004*	10.5	24	35.4	81	75.1	172	19.2	44	10.9	25	-	-	-	-
Multivitamins	27.5	123	27.0	81	28.6	42	0.736*	8.9	11	30.1	37	88.6	109	30.9	38	4.9	6	-	-	-	-
Vitamin B	22.1	99	22.7	68	21.0	31	0.706*	4.0	4	30.3	30	59.6	59	22.2	22	20.2	20	-	-	-	-
Vitamin C	20.8	93	18.7	56	1.0	37	0.112*	10.8	10	19.4	18	87.1	81	20.4	19	4.3	4	-	-	-	-
Antioxidant	12.8	57	10.3	31	17.7	26	0.029*	19.3	11	24.6	14	96.5	55	26.3	15	0.0	0	-	-	-	-
Zink	7.8	35	6.7	20	10.2	15	0.191*	11.4	4	20.0	7	74.3	26	14.3	5	17.1	6	-	-	-	-
Vitamin E	4.0	18	2.7	8	6.8	10	0.037*	5.6	1	22.2	4	83.3	15	27.8	5	11.1	2	-	-	-	-
Vitamin K	3.8	17	3.7	11	4.1	6	0.829*	5.9	1	29.4	5	70.6	12	11.8	2	23.5	4	-	-	-	-
Selenium	3.8	17	4.7	14	2.0	3	0.173*	11.8	2	35.3	6	82.4	14	23.5	4	11.8	2	-	-	-	-
Iodine	3.4	15	3.7	11	2.7	4	0.782^	13.3	2	46.7	7	60.0	9	33.3	5	20.0	3	-	-	-	-
Vitamin A	2.9	13	1.7	5	5.4	8	0.035^	23.1	3	15.4	2	84.6	11	23.1	3	15.4	2	-	-	-	-
Other vitamins and minerals	29.8	133	34.3	103	20.4	30	0.003*	-	-	-	-	-	-	-	-	-	-	-	-	-	-
**Other dietary supplements**	**46.4**	**204**	**48.1**	**143**	**42.7**	**61**	0.279*	**6.4**	**13**	**15.2**	**31**	**92.6**	**189**	**24.5**	**50**	**4.9**	**10**	**42.6**	**87**	**4.4**	**9**
Omega 3 fatty acid	29.5	138	29.5	93	29.4	45	0.980*	8.0	11	18.8	26	90.6	125	26.1	36	2.2	3	39.9	55	4.3	6
Cod-liver oil	27.4	121	27.9	83	24.4	38	0.731*	5.8	7	10.7	13	95.9	116	23.1	28	5.8	7	52.1	63	2.5	3
**Total**	**76.6**	**340**	**78.0**	**234**	**73.6**	**106**	0.307*	**13.2**	**45**	**35.6**	**121**	**85.6**	**291**	**27.4**	**93**	**16.2**	**55**	**45.0**	**153**	**5.9**	**20**

* Pearson chi-square test; ^Fisher exact test

** multiple choice; - data not collected

Cod-liver oil and omega 3 fatty acids were also commonly used (27.4% and 29.5%, respectively), primarily to strengthen the body and the immune system (95.9% and 90.6%). Some participants (23.1% and 26.1%) used these supplements to enhance their quality of life. From the users of cod-liver oil, three individuals (2.5%) experienced mild adverse effects, while six (4.3%) experienced adverse effects from omega 3 fatty acid. Of these, all adverse effects from omega 3 fatty acid were mild, with only one exception which was moderate.

The use of dietary supplements was positively associated with middle age (51–64 years), female genital cancer, cancer treatment, and late and long-term effects of cancer (Table 1). Participants living with their children and those suffering from malignant melanoma were less likely to use dietary supplements (Table 1).

### Disclosure and information gathering regarding dietary changes and supplement use

Generally, approximately half of the participants (51.1%) disclosed their use of dietary modalities to healthcare professionals, with a higher rate of disclosure for dietary supplements (58.2%) compared to dietary changes (44.6%, Table 4). The primary healthcare professionals to whom these disclosures were made were family physicians (24.5%) and oncologists (19.5%).

**Table 4:** Disclosure of dietary changes and supplement usage and gathering of information

	Total dietary changes and supplements		Modifications to exicting diet		Special diets		Total dietary changes		Vitamines and minerals		Other dietary suplements		Total dietary supplements
**Comunication**	**% (n = 456)**		%	*n* = 364	%	*n* = 191	**% (n = 373)**		%	*n* = 313	%	*n* = 204	**% (n = 340)**
*Disclosure*	**51.2**		*43.4*	*158*	*46.8*	*89*	***44.6***		*66.8*	*209*	*45.1*	*92*	***58.2***
Family physician	**24.5**		16.2	59	18.9	*36*	***17.1***		39.9	125	21.1	43	***32.5***
Oncologist	**19.5**		16.5	60	17.4	*33*	***16.8***		26.2	82	16.7	34	***22.4***
Nurse	**6.3**		6.9	25	7.3	14	***7.0***		6.1	19	4.9	10	***5.6***
Other healthcare providers	**6.4**		2.2	8	21.1	*40*	***8.7***		2.9	9	5.4	11	***3.9***
*Non-disclosure*	**48.9**		56.6	206	53.2	*102*	***55.4***		33.2	104	54.9	112	***41.8***
**Information**													
*Gathered/recieved information*	**85.6**		*78.8*	*287*	*88.0*	*168*	***82.0***		*92.0*	*288*	*85.8*	*175*	***89.6***
Internet/media	**39.5**		40.7	148	52.1	*100*	***44.6***		29.7	93	40.7	83	***34.0***
Healthcare providers	**33.8**		30.2	110	24.7	*47*	***28.3***		52.7	165	19.6	40	***39.7***
Family and friends	**20.0**		17.3	63	24.7	*47*	***19.9***		15.0	47	27.9	57	***20.1***
Other	**16.4**		16.8	61	22.0	42	***18.6***		10.2	32	20.1	41	***14.1***
*Did not seek/receive information*	**14.4**		21.2	77	12.0	23	***18.0***		8.0	25	14.2	29	***10.4***

Information was more frequently sought for dietary supplements (89.6%) than dietary changes (82%), especially for vitamins and minerals (92%), which were often obtained from healthcare providers (52.7%). For the other modalities were internet the main source of information (Table 4).

## Discussion

### Main findings

Among the participants, the majority (97%) reported making changes to their diet and/or incorporating dietary supplements in response to their cancer diagnosis, with the primary goal of strengthening their body and immune system. Nearly half of the participants reported that they found these changes beneficial and openly discussed them with their healthcare providers. Adverse effects were reported by only a few participants. Information about dietary changes and supplements was predominantly obtained from the internet or healthcare providers.

### Dietary changes

A high prevalence of dietary changes found in the present study is consistent with findings in a Dutch study where approximately one third of colorectal cancer survivors reported modifying their diet after their cancer diagnosis [14]. In contrast to the present study, the main motive for the dietary changes among the colorectal cancer survivors was to prevent recurrence of cancer. This difference in motive for the dietary changes found in the present study compared to the Dutch study may reflect the results of recent research suggesting that a Mediterranean diet is specifically associated with a lower risk for colorectal cancer. Studies conducted in Italy [36, 37], also revealed a substantial proportion of cancer patients who had adopted a healthier diet following their cancer diagnosis. However, the prevalence of dietary changes was somewhat higher in the present study compared to the Italian and Dutch studies. As in the current study were increased intake of fruit, and reduced intake of red meat, sugar, alcohol, and dairy products among the most commonly changes in Italy [37, 38].

The higher prevalence of dietary changes found in this present study might reflect a growing recognition of the important role of diet and lifestyle factors in cancer prevention. Efforts are also being made to increase public awareness and education on this topic [39–41], including open theme meetings about cancer and diet held by the Norwegian Cancer Society and NAFKAM- Norway’s National Research Center in Complementary and Alternative Medicine - at 15 different locations in Norway two years prior the current study.

Previous Norwegian studies have also identified dietary changes made in response to a cancer diagnosis, with breast cancer and colorectal cancer survivors reported to have modified their diet during their survivorship [33, 42]. In line with the current study, an increase in fruit intake was among the most commonly observed dietary changes, although early-stage breast cancer patients reported a decrease in vegetable consumption [33]. Consistent with our findings, reduced intake of red meat, dairy products, and alcohol were noted; however, contrary to our results, no reduction in sugar intake was reported [33]. The variation in sugar reduction could potentially be attributed to the composition of the earlier study, which exclusively involved female cancer patients, whereas the current study encompassed both male and female participants. Given that males typically exhibit a greater consumption of sugar compared to females [43], it is conceivable that the current cohort necessitated a more pronounced reduction in sugar intake. It’s worth noting that excessive sugar consumption is now acknowledged as a significant contributing factor to obesity; being overweight or having obesity are linked with a higher risk of getting several types of cancer [44].

Additionally the participants were less inclined to make dietary changes overall [33]. The reason for this might be twofold: Firstly, the changes were confined to the last 12 months, and secondly, to be reported as a valid result, the changes needed to be substantial [33].

Importantly, the results presented here, as well as in other studies, highlight the willingness and ability of individuals with cancer to make changes in their diet in accordance with state-of-the-art lifestyle recommendations [7, 9]. However, while the most common change found in the present study was an increased intake of fruits and vegetables, another Norwegian study revealed that only 7.5% of Norwegian cancer patients in northern Norway adhere to the official recommendations of consuming five portions of fruits or vegetables daily [45]. This implies that despite many individuals increasing their intake of fruits and vegetables, the majority still do not meet the recommendations set by health authorities. Regarding alcohol intake, our findings revealed that 43% reported cutting back on alcohol, which aligns with the results of the same study, where 88% of the participants adhered to the recommendations for alcohol consumption (< 10 g per day for women and < 20 g per day for men) [45]. Adherence to lifestyle recommendations is recognized to be challenging. As highlighted in the European Association of Preventive Cardiology clinical consensus document, achieving optimal adherence to lifestyle prevention likely necessitates concerted efforts from various angles, including the patient, the disease, the healthcare provider, the therapy, and the healthcare system [46].

#### Reasons for dietary changes

According to the participants in the present study, most dietary changes were made to strengthen the body and the immune system or to improve the overall quality of life. These findings align with a recent review, which indicated that nutritional interventions may enhance quality of life in general, with specific improvements observed in global health, social function, appetite, cognitive function, and reductions in nausea and fatigue, while also increasing appetite [20]. The most reported dietary changes for this purpose were the reduction of carbohydrates and sugar, made by more than half of the participants. This finding aligns with a Malaysian study, where 53% of the participants also reduced sugar intake resulting in improved emotional function and reduced fatigue [47].

While the majority of the participants altered their diet to enhance their body’s strength, boost their immune system, or improve their overall quality of life, some also expressed the intention of using dietary changes as a potential means to treat or prevent the progression of cancer. While the importance of a healthy diet in cancer prevention is well recognized [4–6, 48], participants in this study primarily associated this concept with the utilization of special diets, rather than moderating existing diet.

#### Adverse effects of dietary changes

Although malnutrition might follow some extreme special diets, few (10%) reported adverse effects of special diets, mostly from juice diets, ketogenic diets, and fasting. More people (12%) reported adverse effects from moderating existing diets, mainly from cutting back on dairy products, carbohydrates, and sugar. Low-carbohydrate, ketogenic diets have received considerable research attention in recent years, and are being applied in various therapeutic contexts, including cancer [49].

According to the Nordic Nutrition Recommendations [50], it is important to ensure a good fatty acid composition in the diet– which means to replace saturated fatty acids with more beneficial unsaturated fatty acids [50]. A common concern physicians have with the ketogenic diet is its effect on blood lipids and lipoproteins, and more broadly, its effect on cardiovascular disease risk factors [51]. A recent study on women with ovarian and endometrial cancer found; however, that a ketogenic diet had no adverse effects on blood lipids [49].

Fasting and fasting-mimicking diets represents a potentially promising strategy to increase treatment efficacy, prevent resistance acquisition and reduce adverse effects when combined with chemotherapy and immunotherapy [52]. Although normally safe, fasting is rarely tolerated by patients [52] and can itself cause adverse effects such as seizure [53].

### Use of dietary supplements

A large European comparative study [27] found that the use of dietary supplement varied markedly in frequency and type across countries, probably due to cultural patterns. More frequent use of dietary supplement was found in Northern compared to Southern Europe and was higher among women than among men [27]. This is in accordance with our finding of high use of dietary supplements in Norway and more so among women. The European study revealed that oil-based supplements, like cod-liver oil were much used in Norway, Denmark, and the United Kingdom. The high use of cod-liver oil found in the present study may be attributed to the strong tradition of cod-liver oil consumption in Norway, along with recommendations from Norwegian health authorities advocating for its daily intake to prevent vitamin D deficiency from an early age [27]. Due to the short daylight hours in wintertime and the absence of sun for months in the northernmost part of the country, preventing vitamin D deficiency has been and remains crucial. Cod-liver oil, readily available due to the abundant fish resources off the coast of Norway, has played a significant role in this aspect. However, despite being frequently used in the current study, vitamin D supplements were found to be more popular, likely due to the distinctive taste of cod-liver oil.

#### Reasons for use of dietary supplements

Although some studies have shown that higher intake of vitamin D is associated with a reduced risk of colorectal cancer [54–57], the results are inconclusive [58]. In the present study, vitamin D and cod-liver oil were mainly used, in the belief, to strengthen the body and the immune system and not to prevent recurrence of cancer, although whole year daily use of cod liver oil has been associated with lower risk of death in patients with solid tumours and in lung cancer patients [59]. To strengthen the body and the immune system was also the main reason for use of dietary supplements in general, in accordance with a Dutch study finding that dietary supplements were used mainly to improve health and prevent disease in general [6].

#### Adverse effects of dietary supplements

Although few participants in the current study experienced adverse effects from dietary supplements, there is evidence suggesting several potential interactions and adverse reactions [60]. Among them are vitamin A, C, D3 and E shown to interact with cancer therapies like Doxorubicin (vitamin C), cisplatin (vitamin C), vincristine (vitamin C), methotrexate (vitamin C), Imatinib (vitamin A, C, D3 and E), Bortezomib (Vitamin C), and radiation therapy (vitamin D) [60]. This is of great concern when this study revealed that only 26% discussed their use of vitamins and minerals with their oncologist, and 33% did not discuss the use of any of these modalities to any healthcare providers.

### Disclosure of dietary changes and supplement use and gathering of information

Satisfactory nutrition can be decisive for further chemotherapy treatment. Consequently, access to information about nutrition and supplements becomes essential in tumor-reducing therapies and is an integral part of the guidelines provided by the Norwegian Directorate of Health [61]. However, it is worth noting that some patients may also resort to additional dietary supplements.

In our survey, a significant majority of participants (85.6%) actively sought information about dietary changes and supplement use from various sources, including the internet (39.5%), healthcare providers (33.8%), and family and friends (20%). Moreover, more than half of the participants (51.2%) disclosed such use to healthcare providers, with the highest disclosure made of vitamins and minerals to family physicians (39.9%) and oncologists (26.2%). Non-disclosure was reported by 48.9% of the participants.

Low disclosure of dietary supplement use has also been reported in other studies [62, 63]. There are various reasons for non-disclosure, including oncologists not inquiring, patients perceiving it as irrelevant, concerns about the oncologist disapproval, lack of knowledge to discuss such matters [62], and communication barriers between oncologists and patients [63]. Given that certain vitamins/minerals and other dietary supplements may interact with conventional cancer treatment [60] this low disclosure rate is a matter of significant concern.

Regarding dietary changes, 28.3% of participants made their changes based on advice from healthcare providers, with 16.8% specifically discussing these dietary changes with their oncologist. This finding aligns with an Italian study, which reported that 24.4% of participants collected information about dietary changes from their oncologist [38]. Previous research conducted in Norway revealed that 54% of cancer patients expressed a need for dietary guidance and/or nutritional treatment during their cancer treatment, whereas only 25% of them actually received such assistance [64]. However, a concerning finding in our study is that 55.4% of participants did not have any discussions regarding dietary changes with any healthcare professionals. This lack of communication is particularly concerning when considering special diets. This highlights the apparent need for clinical nutritionists in municipal healthcare services in Norway, a requirement well recognized and documented [65] as both nurses and doctors have limited training in nutrition during their education. Despite this, the number of positions for clinical nutritionists in the municipal health service increased by only 10 man-years from 2016 to 2020 [16]. In countries where comparisons are natural, the establishment of clinical nutrition expertise in municipalities has made significant progress. For instance, in Sweden, the number of positions for dietitians (equivalent to Norwegian clinical nutritionists) has experienced a substantial increase due to changes in health and social legislation, effective from 2019. These changes aim to provide patients with quicker access to the right skills. Similarly, in the Netherlands, the presence of clinical nutritionists in municipalities now rivals that of hospitals. In England, the National Health Service announced a change in funding arrangements in the spring of 2020, aimed at ensuring and strengthening access to clinical nutritionists in the general practitioner service [66].

Considering the lack of evidence of benefits of special diets and potential malnutrition following strict diets, oncologists should engage more in counselling cancer patients on such diets [67] as most of the changes were made based on information on the internet. The use of internet for such information is not typical for Norwegian cancer patients but are also found elsewhere [38]. Although information about dietary changes can come from reliable sources such as CAM Cancer, national information sites and cancer society’s/cancer hospitals, information is also available from private operators, without expertise in cancer [68].

To enhance this communication gap and improve clinical practice, healthcare providers should be offering training in these modalities. Such a program should not be too ambiguous. Research has demonstrated [69, 70] that a short internet program about the safety of dietary modalities and supplements demonstrated significant and sustained improvements in knowledge, confidence, and communication practices. To get reliable information about the benefit and safety of dietary modalities for cancer, clinicians and individuals with cancer may get helpful information from communication and information tools like CAM cancer [71]. However, further investigation is warranted to determine if the use of such tools can effectively increase disclosure among individuals with cancer and enhance knowledge of these modalities among clinicians. By fostering increased dialogue about diet between patients and healthcare professionals and promoting the use of reliable dietary information, it may also be possible to prevent some individuals from resorting to non-evidence-based and potentially harmful diets and supplements.

### Implication of the findings

The current findings reinforce previous research indicating a strong interest among individuals with cancer to make dietary adjustments or interventions. However, despite this interest, previous research has suggested that individuals do not follow the recommended guidelines provided by healthcare authorities, at least not in arctic Norway [45]. The high level of interest in dietary interventions reported here, coupled with the latest dietary recommendations for secondary cancer prevention (World Cancer Research Fund International [39], European codex [9]), highlights the importance of addressing issues related to adherence to preventative dietary lifestyle interventions among cancer survivors.

This study also reveals that individuals rely heavily on the internet and media as their primary sources of information about dietary interventions and supplements, rather than healthcare professionals and evidence-based information from guidelines and other reliable sources like CAM Cancer. Although it is uncertain what types of internet or media sites are used, it is crucial to explore this issue further, as reliable information is essential.

### Strengths and limitations

The study boasts several strengths, including its adequate study power and high response rate. Additionally, the studied population encompasses a diverse range of cancer sites and diagnoses, representing various geographical regions across Norway (both urban and rural), and mirrors the age distribution seen among adult cancer survivors in the country. A significant advantage of this study is that it was conducted outside a hospital setting, allowing for participation from a broader range of cancer survivors beyond those undergoing active anti-cancer treatment.

However, the study should be interpreted considering certain limitations. A key limitation is that the respondents may not fully represent the entire population of cancer survivors in Norway, particularly due to a higher proportion of female participants compared to the proportion of female cancer patients in general (67% vs. 46%). To address this bias, we have partially presented the results by gender. Another potential limitation is the presence of social bias, which could have led some participants to overreport positive dietary changes. On the other hand, it is possible that some of the reported changes occurred many years ago, leading to recall bias and underreporting of changes made and special diets used. This might also be applicable to the use of dietary supplements, resulting in potential underreporting. Additionally, the fact that the dietary changes were self-reported and not limited to changes meeting dietary recommendations might have contributed to a more positive impression of the willingness to make dietary changes among the cancer survivors in this study. In contrast, another Norwegian study revealed that only 7.5% of cancer survivors adhered to the recommendations of consuming five portions of fruit/vegetables a day [26] although 56% of the participants in the present study reported increased intake of fruits and vegetables.

### Conclusion

This study highlights that most individuals affected by cancer make dietary adjustments. It also emphasizes the importance of addressing adherence to dietary recommendations and using reliable sources of information including guidance by clinical nutritionists. Additionally, the study highlights the potential, yet currently underutilized, role of healthcare professionals in initiating dialogues about dietary interventions to address any unmet needs of patients. Such proactive engagement may contribute to the promotion of reliable sources of information and the prevention of non-evidence-based and potentially harmful diets or supplement adoption.

## Data Availability

The dataset on which this paper is based has not been deposited in any repository. However, all datasets and materials utilized in this study are accessible from the corresponding author upon reasonable request. Interested parties requesting access to the data must, however, be prepared to adhere to Norwegian privacy regulations to ensure compliance with data protection standards.

## Abbreviations

CAM: Complementary and Alternative Medicine
NCS: Norwegian Cancer Society
I-CAM-Q: International Questionnaire to Measure Use of Complementary and Alternative Medicine
NAFKAM: National Research Center in Complementary and Alternative Medicine
NSD: Norwegian Centre for Research Data
